# Workers’ Compensation Claim Rates and Costs for Musculoskeletal Disorders Related to Overexertion Among Construction Workers — Ohio, 2007–2017

**DOI:** 10.15585/mmwr.mm7016a1

**Published:** 2021-04-23

**Authors:** Harpriya Kaur, Steven J. Wurzelbacher, P. Tim Bushnell, James W. Grosch, Chih-Yu Tseng, Juliann C. Scholl, Alysha R. Meyers, Michael Lampl

**Affiliations:** ^1^Division of Science Integration, National Institute for Occupational Safety and Health, CDC; ^2^Division of Field Studies and Engineering, National Institute for Occupational Safety and Health, CDC; ^3^Office of the Director, National Institute for Occupational Safety and Health, CDC; ^4^Ohio Bureau of Workers’ Compensation, Columbus, Ohio.

Overexertion is a leading cause of work-related musculoskeletal disorders (WMSDs) among construction workers. Nearly 90% of construction jobs require manual handling of materials for approximately one half of the worker’s time (*1*). In 2015, overexertion from lifting and lowering materials caused 30% of WMSDs among construction workers; overexertion involving pushing, pulling, holding, carrying, and catching materials caused an additional 37% of WMSDs (*1*). This study examined the rate and cost of WMSD claims from overexertion among Ohio construction workers during 2007–2017. Workers’ compensation claims related to overexertion that were submitted to the Ohio Bureau of Worker’s Compensation (OHBWC) by workers in the construction industry for injuries and illnesses occurring during 2007–2017 were analyzed. Rates and costs of allowed claims were measured by age group. Workers aged 35–44 years experienced the highest claim rate: 63 per 10,000 full-time employees (FTEs) for WMSDs from overexertion. However, claims by workers aged 45–54 years and 55–64 years were more costly on average and resulted in more days away from work. Ergonomic design improvements and interventions are needed to ensure that the majority of construction workers can safely perform jobs throughout their careers. Age-specific WMSD prevention and risk communication efforts also might be helpful.

From 1985 to 2015, the average age of construction workers increased from 36 years to 42.5 years (*2*). As workers age, they become more susceptible to losing muscle mass and strength (*3*). These and other age-related physical changes can affect workers’ ability to perform physically demanding tasks, their vulnerability to WMSDs, and their ability to recover from WMSDs. As the U.S. workforce grows older, understanding the age-specific health and safety needs of workers is critical, especially in hazardous and physically demanding industries such as construction.

Data for this report came from workers’ compensation claims for WMSDs filed by employees of state-insured private industry employers in Ohio* during 2007–2017. Ohio is the most populous of the four states (North Dakota, Ohio, Washington, and Wyoming) that have exclusive state-run workers’ compensation systems. Ohio insures approximately two thirds of the state’s workforce. In Ohio, only large employers (usually those with ≥500 employees) may self-insure. Lost-time claims (those with ≥8 days away from work) and medical-only claims (only medical treatment expenses paid and ≤7 lost work days) were analyzed. Claim data fields included employer information, worker age and gender, claim cost, lost work days, diagnosis billing codes (*International Classification of Diseases, Ninth Revision, Clinical Modification* [ICD-9-CM]), and a free-text narrative that described how the injury or illness occurred.

All claim narratives were auto-coded using two algorithms (*4*,*5*). The first algorithm identified claims that met the U.S. Bureau of Labor Statistics (BLS) case definition for a WMSD.^†^ The second algorithm identified a subset of WMSD claims that met the BLS Occupational Injury and Illness Classification System definition for overexertion involving an outside source.^§^ High-cost (95th percentile or higher) claims and lost-time claims with low estimated probabilities of an accurately auto-coded diagnosis were manually reviewed by expert coders. When a claim had multiple ICD-9-CM diagnosis codes, an OHBWC algorithm was used to identify the diagnosis most likely to keep the worker off work for the longest period.

Worker’s compensation claims were linked to Ohio unemployment insurance data to determine employer industry and employee count using methods developed by previous studies (*6*). The construction industry was identified by North American Industry Classification System code 23.^¶^ American Community Survey** yearly data contain information on number of hours worked per construction worker and were used to convert number of employees to number of FTEs. American Community Survey data also were used to estimate the percentage of the Ohio construction worker population within each age group, which was used to calculate age-specific rates. Cumulative claim rates were calculated by dividing the sum of the yearly claim counts by the sum of the yearly estimated FTEs for 2007–2017.

The most recently estimated total costs^††^ were used to calculate cost per claim and cost per FTE by age group. The number of lost work days associated with each claim was the number recorded as of June 30, 2019.^§§^ For each age group, the percentage of claims that were lost-time claims and the percentage of lost-time claims with ≥100 lost work days were calculated as indicators of claim severity. SAS (version 9.4; SAS Institute) was used to conduct all analyses. This activity was reviewed by CDC and was conducted consistent with applicable federal law and CDC policy.^¶¶^

During 2007–2017, OHBWC accepted 10,347 claims*** from construction workers for WMSDs resulting from overexertion. The rate of WMSD claims per 10,000 FTEs from overexertion among construction workers was highest among those aged 35–44 years (63.0), followed by claim rates among those aged 45–54 years (59.6) and those aged 25–34 years (55.5). The relationship between WMSD rate and age differed by diagnosis category. The claim rate for spinal disc disorders was highest among those aged 35–44 years (4.7) and 45–54 years (4.5), as was the rate of upper extremity sprains (18.5 among those aged 35–44 years and 18.6 among those aged 45–54 years). The rate of back sprain claims was highest among those aged 25–34 years (26.5) and 35–44 years (24.6) (Figure).

**FIGURE:**
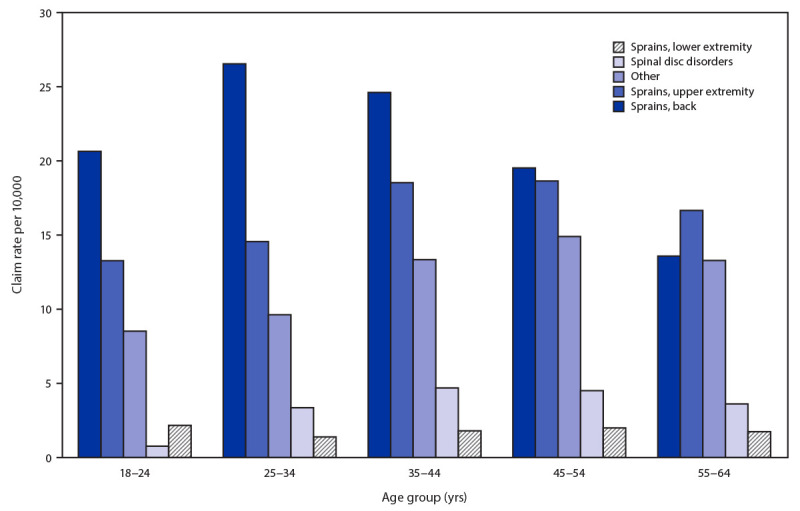
Rate of work-related musculoskeletal disorder claims from overexertion per 10,000 full-time employees among construction workers, by diagnosis category* and age group — Ohio, 2007–2017 * Among these, 63% were single diagnoses. For multiple diagnoses, an algorithm was used to identify the diagnosis most limiting the ability to return to work.

The severity of WMSDs, as measured by the percentage of claims classified as lost-time (≥8 lost work days), increased with age, peaking among those aged 55–64 years (Table 1). The percentage of lost-time claims with ≥100 work days lost was highest among those aged 45–54 years and lowest among those aged 18–24 years. Cost per claim was highest among those aged 45–54 years ($25,932) and 54–64 years ($25,572). Cost per FTE was highest among those aged 45–54 years ($154.56) (Table 2). The relationship between cost and age differed by diagnosis category; for example, cost per FTE for back and lower extremity sprains peaked among those aged 35–44 years and 25–34 years, respectively, whereas spinal disc disorders and upper extremity sprain costs per FTE peaked among those aged 45–54 years and 55–64 years, respectively.

**TABLE 1:** Age-specific numbers and rates of work-related musculoskeletal disorder (WMSD) claims from overexertion among construction workers — Ohio, 2007–2017

Claim	No. of claims, by age group (yrs)				
	18–24	25–34	35–44	45–54	55–64
**Total (all overexertion WMSDs)**	**879**	**2,570**	**3,004**	**2,746**	**1,148**
**Diagnosis category**					
Spinal disc disorders	15	156	224	208	85
Upper extremity sprains	257	674	884	859	391
Lower extremity sprains	42	65	86	92	41
Back sprains	400	1,229	1,174	900	319
Other overexertion WMSDs*	165	446	636	687	312
**No. of FTEs**	193,702	463,026	476,862	460,729	234,751
**Claims per 10,000 FTEs**					
**Total (all overexertion WMSDs)**	**45.4**	**55.5**	**63.0**	**59.6**	**48.9**
**Diagnosis category**					
Spinal disc disorders	0.8	3.4	4.7	4.5	3.6
Upper extremity sprains	13.3	14.6	18.5	18.6	16.7
Lower extremity sprains	2.2	1.4	1.8	2.0	1.8
Back sprains	20.7	26.5	24.6	19.5	13.6
Other overexertion WMSDs*	8.5	9.6	13.3	14.9	13.3
**Percentage of claims with high number of lost work days (≥8 days, ≥100 days)**					
Lost-time claims (≥8 lost work days) as percentage of all claims	18.0	23.8	32.0	38.6	40.5
Percentage of lost-time claims with ≥100 lost work days	31.6	40.6	43.6	45.0	42.4

**Abbreviation:** FTE = full-time employee.

* Other overexertion WMSDs include carpal tunnel syndrome, diseases of musculoskeletal and connective tissues, hernia of abdominal cavity, soft tissue/enthesopathy, other sprains, dislocation, spinal osteoarthritis, diseases of the nervous system and sense organ, injury to nerves and spinal cord, knee derangement, other joint disorders, and symptoms, signs, and ill-defined conditions, not elsewhere classified.

**TABLE 2:** Age-specific costs of work-related musculoskeletal disorder (WMSD) claims from overexertion among construction workers, by diagnosis — Ohio, 2007–2017

Claim	Cost per claim ($),* by age group (yrs)				
	18–24	25–34	35–44	45–54	55–64
**Medical incurred cost (all diagnoses)**	2,031	5,893	9,611	11,471	10,446
**Medical cost by diagnosis category**					
Spinal disc disorders	22,272	58,169	66,306	70,422	54,379
Upper extremity sprains	1,462	3,195	6,216	7,783	9,095
Lower extremity sprains	1,269	4,240	3,229	2,218	1,604
Back sprain	1,102	1,369	2,187	1,918	1,776
Other overexertion WMSDs†	3,522	4,391	8,927	11,987	10,198
**Indemnity incurred cost (all diagnoses)**	1,461	5,918	10,749	14,461	15,126
**Indemnity incurred cost by diagnosis category**					
Disc disorders	26,127	62,991	77,538	90,859	100,709
Upper extremity sprains	717	2,712	5,147	7,500	10,593
Lower extremity sprains	586	3,352	1,507	845	1,322
Back sprain	535	1,088	2,474	3,764	1,287
Other overexertion WMSDs†	2,847	4,483	11,537	15,869	13,454
**Total cost (all diagnoses)**	**3,492**	**11,811**	**20,359**	**25,932**	**25,572**
**Total cost by diagnosis category**					
Spinal disc disorders	48,400	121,159	143,845	161,281	155,088
Upper extremity sprains	2,179	5,907	11,362	15,284	19,688
Lower extremity sprains	1,855	7,592	4,736	3,063	2,925
Back sprain	1,637	2,457	4,661	5,682	3,062
Other overexertion WMSDs†	6,369	8,874	20,464	27,857	23,652
**Cost per FTE**					
**Medical incurred cost (all diagnoses)**	9.22	32.71	60.54	68.37	51.09
**Medical Incurred cost by diagnosis category**					
Spinal disc disorders	1.72	19.60	31.15	31.79	19.69
Upper extremity sprains	1.94	4.65	11.52	14.51	15.15
Lower extremity sprains	0.28	0.60	0.58	0.44	0.28
Back sprain	2.28	3.63	5.38	3.75	2.41
Other overexertion WMSDs†	3.00	4.23	11.91	17.87	13.55
**Indemnity incurred cost (all diagnoses)**	6.63	32.85	67.71	86.19	73.97
**Indemnity incurred cost by diagnosis category**					
Spinal disc disorders	2.02	21.22	36.42	41.02	36.47
Upper extremity sprains	0.95	3.95	9.54	13.98	17.64
Lower extremity sprains	0.13	0.47	0.27	0.17	0.23
Back sprain	1.10	2.89	6.09	7.35	1.75
Other overexertion WMSDs†	2.42	4.32	15.39	23.66	17.88
**Total cost (all diagnoses)**	**15.85**	**65.56**	**128.26**	**154.56**	**125.05**
**Total cost by diagnosis category**					
Spinal disc disorders	3.75	40.82	67.57	72.81	56.16
Upper extremity sprains	2.89	8.60	21.06	28.50	32.79
Lower extremity sprains	0.40	1.07	0.85	0.61	0.51
Back sprain	3.38	6.52	11.48	11.10	4.16
Other overexertion WMSDs†	5.43	8.55	27.29	41.54	31.43

**Abbreviation:** FTE = full-time employee.

* Costs were total incurred costs as of June 30, 2019, which include all costs paid up to that time, plus the amount set aside for reserves to pay projected future costs of the same set of claims.

† Other overexertion WMSDs include carpal tunnel syndrome, diseases of musculoskeletal and connective tissues, hernia of abdominal cavity, soft tissue/enthesopathy, other sprains, dislocation, spinal osteoarthritis, diseases of the nervous system and sense organ, injury to nerves and spinal cord, knee derangement, other joint disorders, and symptoms, signs, and ill-defined conditions, not elsewhere classified.

## Discussion

The findings in this report are consistent with those of recent studies indicating that the rate of overexertion-related WMSD claims rise and then fall with increasing age (*7*,*8*). This pattern has at least two explanations. First, older workers might shift to other tasks or jobs with reduced WMSD risks. Second, workers experiencing severe pain might move out of the industry, leaving behind a healthier cohort. A longitudinal study among construction roofers found that the odds of leaving the roofing trade early were eight times higher for workers with WMSDs than for workers without such disorders (*9*). Additional analyses of WMSD rates that include former and current construction workers are needed to determine the actual rates and severity of overexertion-related WMSDs by age group for the construction industry.

The findings in this report are subject to at least three limitations. First, auto-coding methods used to identify WMSD claims entail some misclassification (*4*,*5*). Misclassification would not be expected to vary by age if claim records are similar in completeness and accuracy across age groups, but if misclassification varies by type of WMSDs, this could bias the comparison of the mix of WMSDs by age group. Overall, the auto-coding methods have been shown to have positive predictive values >85% when compared with manual coding (*4*,*5*). Second, not all work injuries and illnesses result in workers’ compensation claims. For example, one study of six states estimated that workers’ compensation claims accounted for approximately 65% to 95% of work-related lost-time cases (>3 or >7 lost work days) (*10*). Underreporting of WMSDs might differ by age group. Finally, data in Ohio are available only for insured private companies; therefore, the degree to which these results reflect age patterns among large, self-insured employers who do not purchase workers’ compensation policies is uncertain.

WMSDs affect Ohio construction workers of all age groups, but do so differently. As age increases, the severity of WMSDs appears to rise, and the relative frequencies of WMSD types change. This suggests the potential usefulness of targeting some prevention efforts specifically to the needs of older workers. For example, differences between age groups in the rate and severity of specific WMSD types might be communicated to workers and their supervisors to help them focus on the most important risks. Considering the high rates of WMSDs among workers aged 25–44 years, and the fact that construction workers with WMSDs tend to leave the workforce prematurely (*2*), workplace ergonomic design and interventions for workers of all ages should be considered. These measures include modifying tasks, promoting the use of ergonomic tools and equipment, providing training in safe work practices, and other interventions.^†††^^,^^§§§^

**Summary**

**What is already known about this topic?**

Overexertion is the major cause of work-related musculoskeletal disorders (WMSDs) among U.S. construction workers.

**What is added by this report?**

Although the prevalence of workers’ compensation WMSD claims from overexertion among construction workers during 2007–2017 in Ohio was highest among workers aged 35–44 years, the average claim was more costly and resulted in more days away from work among workers aged 45–54 years and 55–64 years.

**What are the implications for public health practice?**

Ergonomic design improvements and interventions are needed to make the workplace safer for workers of all ages. Age-specific WMSD prevention and risk communication efforts also might be helpful.

## Footnotes

* All worker compensation claims were from OHBWC-insured, single- and multiple-location private industry employers. Large employers (usually with ≥500 employees) have the option to self-insure if they meet certain requirements. In 2017, 54% of those employers were estimated to have done so. Owners of sole proprietorships and partnerships do not have to insure themselves but must insure any employees they have. In 2017, 38% of sole proprietorships did not purchase OHBWC coverage.

† This analysis used a definition for musculoskeletal disorders that included Raynaud’s phenomenon, tarsal tunnel syndrome, and herniated spinal discs, similar to the revised BLS case definition (2011 and forward). https://www.bls.gov/iif/oshdef.htm

§ According to the Occupational Injury and Illness Classification System, overexertion involving outside sources (code 71), “applies to cases, usually non-impact, in which the injury or illness resulted from excessive physical effort directed at an outside source of injury or illness.” Typically, an outside source refers to anything or any person against which the worker exerted force during the bodily motions thought to have caused the WMSD. This could involve, for example, pushing, pulling, or lifting. https://www.bls.gov/iif/osh_oiics_2010_2_4.pdf

¶
https://www.naics.com/six-digit-naics/?code=23

**
https://www.census.gov/programs-surveys/acs

†† Costs were total incurred costs (not adjusted for inflation) as of June 30, 2019. Those include all costs for medical treatments and indemnity (partial lost wage replacement) paid up to that time, plus the amount set aside for reserves to pay projected future costs of the same set of claims. The costs of some claims for WMSDs occurring during 2007–2017 had not all been paid as of 2019 because some workers were still receiving medical care for the WMSD or were still entitled to indemnity payments to compensate for ongoing or recurring loss of work.

§§ The eventual total number of days lost is not known for those claims for which work days were lost after 2019.

¶¶ 45 C.F.R. part 46, 21 C.F.R. part 56; 42 U.S.C. Sect. 241(d); 5 U.S.C. Sect. 552a; 44 U.S.C. Sect. 3501 et seq.

*** This total excludes 95 claims for which age was missing.

†††
https://www.cpwr.com/research/research-practice-library/construction-ergonomic-research-solutions

§§§
https://www.lhsfna.org/index.cfm/occupational-safety-and-health/ergonomics/?PAGENUM=1&WIPECACHE=false
